# Conducting multicenter research in healthcare simulation: Lessons learned from the INSPIRE network

**DOI:** 10.1186/s41077-017-0039-0

**Published:** 2017-02-28

**Authors:** Adam Cheng, David Kessler, Ralph Mackinnon, Todd P. Chang, Vinay M. Nadkarni, Elizabeth A. Hunt, Jordan Duval-Arnould, Yiqun Lin, Martin Pusic, Marc Auerbach

**Affiliations:** 1 Department of Pediatrics, Alberta Children’s Hospital, KidSim-ASPIRE Research Program, Section of Emergency Medicine, University of Calgary, 2888 Shaganappi Trail NW, Calgary, AB Canada T3B 6A8; 2 Division of Pediatric Emergency Medicine, Columbia University Medical School, 3959 Broadway, CHN-1-116, New York, NY 10032 USA; 3 Department of Paediatric Anaesthesia and NWTS, First Floor Theatres, Royal Manchester Children’s Hospital, Hathersage Road, Manchester, UK M13 9WL; 4 Children’s Hospital Los Angeles, 4650 Sunset Blvd, Mailstop 113, Los Angeles, CA 90027 USA; 5 The Children’s Hospital of Philadelphia, University of Pennsylvania Perelman School of Medicine, 3401 Civic Center Blvd, Philadelphia, PA 19104 USA; 6 Charlotte R. Bloomberg Children’s Center, Johns Hopkins University School of Medicine, 1800 Orleans St, Room 6321, Baltimore, MD 21287 USA; 7 Alberta Children’s Hospital, Cumming School of Medicine, University of Calgary, 2888 Shaganappi Trail NW, Calgary, AB Canada T3B 6A8; 8 Institute for Innovations in Medical Education, 550 First Ave, MSB G109, New York, NY 10016 USA; 9 Section of Pediatric Emergency Medicine, 100 York Street, Suite 1F, New Haven, CT 06520 USA

**Keywords:** Knowledge Translation, Advanced Life Support, Primary Investigator, Feasibility Testing, Site Investigator

## Abstract

Simulation-based research has grown substantially over the past two decades; however, relatively few published simulation studies are multicenter in nature. Multicenter research confers many distinct advantages over single-center studies, including larger sample sizes for more generalizable findings, sharing resources amongst collaborative sites, and promoting networking. Well-executed multicenter studies are more likely to improve provider performance and/or have a positive impact on patient outcomes. In this manuscript, we offer a step-by-step guide to conducting multicenter, simulation-based research based upon our collective experience with the International Network for Simulation-based Pediatric Innovation, Research and Education (INSPIRE). Like multicenter clinical research, simulation-based multicenter research can be divided into four distinct phases. Each phase has specific differences when applied to simulation research: (1) *Planning phase*, to define the research question, systematically review the literature, identify outcome measures, and conduct pilot studies to ensure feasibility and estimate power; (2) *Project Development phase*, when the primary investigator identifies collaborators, develops the protocol and research operations manual, prepares grant applications, obtains ethical approval and executes subsite contracts, registers the study in a clinical trial registry, forms a manuscript oversight committee, and conducts feasibility testing and data validation at each site; (3) *Study Execution phase*, involving recruitment and enrollment of subjects, clear communication and decision-making, quality assurance measures and data abstraction, validation, and analysis; and (4) *Dissemination phase*, where the research team shares results via conference presentations, publications, traditional media, social media, and implements strategies for translating results to practice. With this manuscript, we provide a guide to conducting quantitative multicenter research with a focus on simulation-specific issues.

**Electronic supplementary material:**

The online version of this article (doi:10.1186/s41077-017-0039-0) contains supplementary material, which is available to authorized users.

## Background

Simulation-based research (SBR) addresses either the impact of simulation as an educational intervention or as an investigative methodology to study clinically important questions [1]. Despite the increase in published healthcare simulation research, relatively few studies are multicenter in nature [2]. Many published single-center studies fail to make an impact on educational or clinical practice or have a follow-up multicenter study conducted. An increase in multicenter SBR has the potential to improve the level and quality of evidence to help to inform change that drives patient care and outcomes.

There are many important benefits of collaborative, multicenter research. Multicenter research allows analysis of questions that require larger sample sizes [3], while enabling comparison of effect between sites and providing insight related to generalizability of effect across institutions [4]. Multicenter research promotes capacity, networking, and mentorship by bringing together investigators who share and leverage resources, expertise, and ideas [4–7]. Research networks can support multicenter collaborations by providing infrastructure, site investigators and content experts, and opportunities for dissemination of best practices to and beyond network members [4, 8, 9]. Collaborative research teams involving members from various professions or disciplines incorporate multiple perspectives that introduce new knowledge and concepts to improve healthcare [10]. As a consequence of higher quality research, teams conducting multicenter studies are able to publish in higher impact peer-reviewed journals [5], contributing to a track record of success that leads to more effective dissemination and may support funding for future projects.

The International Network for Simulation-based Pediatric Innovation, Research and Education (INSPIRE) is the world’s largest simulation network focused on improving healthcare outcomes through collaborative, multicenter research [9]. As INSPIRE network investigators, we have reflected on our past successes, challenges, and failures in conducting large, multicenter, simulation-based studies [9, 11–25]. In this manuscript, we provide a guide to conducting quantitative multicenter research with a focus on simulation-specific issues. We hope this guide will help facilitate collaboration and multicenter SBR that will positively impact patient care and clinical outcomes.

**Fig. 1:**
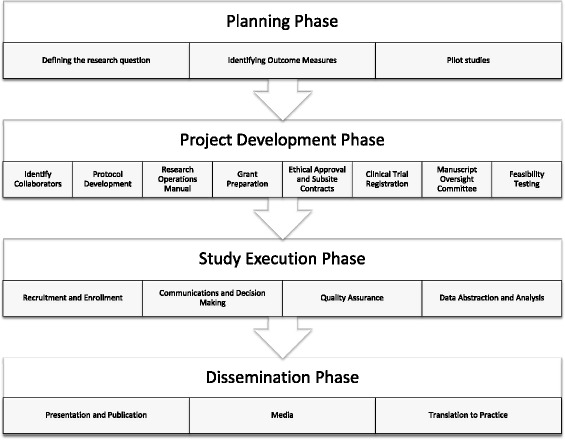
Four phases for planning and conducting multicenter simulation-based research

We identify four key phases to plan and conduct multicenter SBR: (1) Planning, (2) Project Development, (3) Study Execution, and (4) Dissemination (Fig. 1). While some of these phases and the steps within have been described for non-SBR related studies [3, 7, 26, 27], we share a unique perspective as simulation researchers by focusing on simulation-specific issues and related recommendations. For each step of the multicenter research process, we offer a simulation research pearl—a high-yield, simulation-relevant tip that will help investigators successfully complete their multicenter SBR project. Table 1 highlights the differences between clinical trials and simulation-based studies and provides practical tips for investigators to consider when conducting multicenter simulation-based research. To facilitate the research process, we provide a checklist for investigators (Table 2) to use as they progress through the various phases and steps of multicenter simulation-based research.

**Table 1:** Differences between clinical trials and simulation studies: implications for multicenter simulation-based research

Research phase/step	Clinical study	Simulation study	Implications for multicenter simulation-based research
Planning phase			
Identifying outcome measures	Clinical outcomes	Education and/or performance outcomes, clinical outcomes or both	Investigators should ensure data captured is reliable and accurate. Equipment should be calibrated and tested across sites prior to study implementation.
Project Development phase			
Research operations manual	Clinical environment difficult control/standardize	Simulated environment can be standardized to isolate independent variable.	The simulation-based research environment and intervention (if applicable) should be carefully standardized across sites to minimize risk of bias.
Grant preparation	Clinical studies typically have clinical outcomes.	Simulation studies may have T1 or T2 outcomes.	In grants, investigators should outline a chain of causality: describing the potential link between proposed outcomes, often accessible outcomes and the relevant patient outcomes.
Ethical approval and subsite contracts	Clinical studies require full ethical approval.	Some simulation studies may be exempt from full ethical review.	Investigators should share institutional review board comments with collaborators submit an inquiry for ethics exempt status at all sites (when appropriate).
Clinical trial registration	Clearly required for all clinical trials	Unclear if required for simulation studies reporting T1 outcomes.	Investigators should prospectively registering simulation-based studies, thereby eliminating the possibility that a manuscript be rejected due to a lack of trial registration.
Manuscript oversight committee	Clinical journals are most likely target.	Simulation, education, clinical journals may all be possible targets.	Research teams should consider the journal scope, focus, and audience when selecting a journal—and matching this to the study topic, quality, and ideal audience.
Feasibility testing	Clinical environment is variable and controlling variability is difficult.	Simulation environment is variable but control is possible.	Feasibility testing helps identify and minimize sources of variance/potential confounding variables across sites.
Study Execution			
Recruitment and enrollment	Patients are recruited by study personnel.	Participants (healthcare providers or trainees) are recruited by study personnel.	Participants may have different expectations of participation in simulation-based research from site to site. Investigator must ensure that all sites have the appropriate pool of healthcare providers or trainees to recruit as participants.
Data abstraction and analysis	Video review infrequently used as means to collect outcomes	Video review and performance assessment often used to collect outcomes (T1 level)	Feasibility testing should be conducted to ensure quality of audio and video across sites.
			Raters must be trained—allocation videos to raters should be done to avoid bias.
Dissemination			
Publication	Educational content typically not part of clinical trials	Educational content may be published or shared as enduring materials.	Educational interventions developed for simulation-based educational studies can be disseminated through publication to facilitate implementation by educators.

This table describes only the steps in the research process where differences between clinical trials and simulation studies exist. Steps where there are no discernable differences have been left out of the table

**Table 2:** Checklist for Conducting Multicenter Simulation Research

Study Phase & Step	Items to Consider
Planning Phase	
Defining the Research Question	o Have consensus recommendations/guidelines for simulation research been reviewed? o Has a review of the published literature (within and outside of simulation / healthcare) been conducted? o Is the question feasible, interesting, novel, ethical and relevant?
Identifying Outcome Measures	o Is it possible to measure a clinically relevant outcome? o If simulation outcomes are being collected -- have the measurement tool(s) been previously validated? Can you establish a causal link to clinically relevant outcomes? o If technology is being used to collect data, is the data known to be reliable and accurate?
Pilot Study	o Have you conducted/do you plan to conduct a pilot study? o Is a multicenter study needed to answer the question? If so why - for generalizability, sample size? o If the pilot study is complete, have you documented lessons learned and iteratively adapted your work for protocol development of multicenter study?
Project Development Phase	
Identify Collaborators	o Does your research team have expertise in all relevant areas? o Have you considered inviting trainees or junior faculty as collaborators? o Have you considered inviting collaborators and/or content experts from outside the simulation community? o Do you have a clear role for each collaborator?
Protocol Development	o Have all members of your research team had opportunity to contribute to protocol development? o Have external experts and/or peers reviewed your protocol and provided feedback? o Have you reached consensus before finalizing the protocol?
Research Operations Manual	o Does your research operations manual contain all the key elements: consent and recruitment strategy, study team members, study flow, inclusion and exclusion criteria, study methods and data management procedures? o Have you described in detail how to standardize the simulation environment (and intervention if applicable) across sties? o Have you conducted training sessions for other sites (either in person or remotely)?
Grant Preparation	o Is it possible to complete your multicenter study without grant funding? o Have you maximized opportunities for matching/in-kind support? o Have you identified appropriate funding agencies and opportunities that align with your research objectives? o Have you appropriately prioritized high value items in your budget?
Ethical Approval and Subsite Contracts	o Has the lead study site obtained ethics approval? o Has a copy of the approved ethics submission been circulated to collaborators? o Has feedback from prior ethics board reviews been circulated to collaborators? o Has work to execute subsite contracts been initiated early to prevent delays in research? o Have you created a timeline for ethics review for participating sites?
Clinical Trial Registration	o Has the study been registered in a clinical trial registry?
Manuscript Oversight Committee	o Has a manuscript oversight committee been established? o Has the manuscript oversight committee developed a document outlining planned publications with proposed writing teams and target journals? o Have all collaborators received a copy of this document?
Feasibility Testing	o Have all sites conducted feasibility testing? o Have lessons learned from feasibility testing been shared across sites?
Study Execution Phase	
Recruitment and Enrollment	o Is the consent process standardized across sites? o For randomized controlled trials, does the randomization process ensure equal allocation of study groups within sites?
Communication and Decision Making	o Has a plan for frequent, planned communications amongst investigators been established? o Has a clear organizational structure been established and communicated with all collaborators? o Has a research steering committee been established? o Do all sites have a research operations committee? o Is there a strategy to foster collaborative spirit and to build team cohesion? o Is there a plan to use motivational strategies (eg. dashboards)?
Quality Assurance	o Is there a quality assurance plan? o Will there be centralized monitoring and intermittent review of data? o Will there be site visits by the principal investigator or core study team members?
Data Abstraction and Analysis	o When assessment tools are being used, is there a plan to do rater orientation training? o When assessment tools are being used, is there a plan to do rater booster training? o When video review is being used, to avoid bias, can raters be assigned participants that are not from their own site? o Has missing data been analyzed to determine if systematic biases exist?
Dissemination Phase	
Presentation and Publication	o Have abstracts been submitted for presentation at relevant conferences? o Has the research team taken advantage of all potential opportunities for publication? o Has the main study been submitted and published prior to other sub-studies?
Media	o Does the research team have a media dissemination strategy? o Has the research team considered opportunities for dissemination through traditional media, social media and online forums?
Translation to Practice	o Have relevant stakeholders been engaged in efforts to translate results to practice? o Have the results been shared with colleagues within existing networks and societies?
Future work	o Has the team discussed next steps for this research group/question?

### Planning phase

#### Defining the research question(s)

To identify an important research question, investigators can seek guidance from existing research networks that have identified knowledge gaps and developed consensus for the future of simulation research. For example, in 2011, the Society for Simulation in Healthcare and the Society in Europe for Simulation Applied to Medicine conducted an Utstein Style Meeting to set a research agenda for simulation-based healthcare education [28]. The INSPIRE network also conducted a consensus building exercise to define six specific areas of focus to help advance the field of pediatric simulation [9]. The results from consensus meetings serve as a roadmap for future multicenter research projects, while networks may provide feedback to help fine-tune the research question. Defining an objective that is feasible, ethical, and has the potential to positively impact learner and/or patient outcomes should drive the genesis of the research question [29]. Table 3 offers a case study in multicenter research based on a recent INSPIRE multicenter study.

**Table 3:** Case study in multicenter research: challenges and lessons learned

*In this case study, we reflect our experiences conducting the CPRCARES study (Improving Cardiopulmonary Resuscitation with a CPR Feedback Device and Refresher Simulation)—prospective, multicenter, randomized,* 2 × 2 *factorial designed study conducted across 10 INSPIRE network sites* [11]. *The main objective of the study was to determine whether just-in-time CPR training before cardiac arrest, or real-time visual CPR feedback during cardiac arrest, improves the quality of chest compressions during a simulated cardiac arrest scenario.*
Planning phase
**Defining the research question(s)**: We had two research questions we wanted to answer. We decided to conduct a 2 × 2 factorial designed study, allowing us to answer both questions in one study. *Lesson learned: Multicenter research allows for sufficient sample size to conduct factorial design studies.* **Outcome measures:** We wanted to collect CPR quality data from both the CPR feedback device and the mannequin. We needed a mannequin permitting chest compressions to >5 cm (as CPR guidelines recommend a depth of 5–6 cm), so the manufacturer provided custom-made chest springs allowing for a maximum compression depth of 7 cm. The chest springs were installed in mannequins across all 10 sites. *Lesson learned: If the mannequin is to be used to collect data, it must have the appropriate functional fidelity.* **Pilot studies:** We did not do a pilot study and instead used results from prior clinical studies to inform our power calculation. Without the pilot study, we were less prepared for the multicenter center and as a result forced to troubleshoot many issues during feasibility testing and during recruitment that could have been avoided. *Lesson learned: Pilot studies not only help to inform sample size/power calculations but also provide value experience to help inform the design of the multicenter research protocol.*
Project Development phase
**Identify collaborators:** Just-in-time CPR training was one of the interventions so we excluded sites where just-in-time CPR training was occurring. Unfortunately, this meant investigators from recruitment sites did not have prior experience with just-in-time CPR training. To address this issue, we invited collaborators with experience using just-in-time CPR training to help develop the protocol. *Lesson learned: Inviting collaborators who have prior experience with the intervention is important for protocol development.* **Protocol Development:** We presented the proposed study at an INSPIRE annual meeting and receive great feedback that was incorporated into the protocol. Unfortunately, the protocol revisions led to unexpected delays. *Lesson learned: The research timeline should appropriately budget for time to revise the protocol after receiving feedback.* **Research operations manual:** We trained our confederates in a very thorough manner and measured their compliance with the tightly scripted confederate roles. *Lesson learned: Confederate compliance with pre-scripted behaviors can be very high if they are trained in a rigorous manner.* **Grant preparation:** Our study scenario was a case of cardiac arrest progressing from one rhythm to another. The simulated clinical environment was also standardized across sites. Reviewers questioned the generalizability of our findings across different institutions (where clinical environments differ) and across different patient presentations of cardiac arrest (i.e., different rhythms). *Lesson learned: While standardization is a strength of simulation research, it may also be perceived as a limitation when it comes to generalizability.* **Ethics approval and subsite contracts:** We submitted several ethics amendments for sub-study ideas that emerged during discussions. This led to a significant delay. *Lesson learned: Ensure that all ideas for possible sub-studies have been discussed and incorporated into the research proposal prior to ethics submission.* **Clinical trial registration:** We were asked to provide a clinical trial registration number upon submission of the manuscript for publication. *Lesson learned: Ensure your study is prospectively registered in a clinical trial registry prior to initiation of recruitment.* **Manuscript oversight committee:** We had planned for several sub-studies to be published as separate manuscripts. Writing groups were assigned by the manuscript oversight committee which resulted in no conflicts between investigators related to authorship order. *Lesson learned: Transparency and clarity is key to prevent conflict between investigators for potential publications resulting from multicenter research projects.* **Feasibility testing:** Despite feasibility testing, we still had one or two sites that submitted videos with very poor audio quality—making it difficult to use those videos in certain analysis. *Lesson learned: Have all sites test audio and video quality before each recruitment session.*
Study Execution phase
**Recruitment and enrollment:** Some sites fell short of their recruitment quota due to lack of available participants. Ongoing local studies at some sites with related interventions and outcomes limited the number of possible participants at those sites. *Lesson learned: Collect an inventory of ongoing related studies at all potential sites and consider these studies when estimating size of potential participant poll.* **Communications and decision-making:** We had regular conference calls and annual face-to-face meeting that helped keep the study on track. Unfortunately, these calls dropped off after the study was complete, making communication more challenging during the dissemination phase. *Lesson learned: Continue regular conference calls (and consider face to face meetings) during dissemination phase of research.* **Quality assurance:** We instituted centralized monitoring of videos in this study, allowing us to identify poor video quality at one site early on in recruitment. This resulted in a fix when improved video quality for subsequent sessions. *Lesson learned: Centralized monitoring of data and videos is a critical quality assurance measure.* **Data abstraction and analysis:** We trained raters to use a tool to assess clinical performance by viewing videos of the simulated cardiac arrest. Videos were not available for rating until 6 months after the rater training, necessitating repeat rater training and re-calibration. *Lesson learned: Timing of rater training is critical. Ideally, raters should be trained and calibrated immediately prior to rating performance.*
Dissemination phase
**Presentation and publication:** We aimed to publish the main study first and sub-studies shortly thereafter. One or two sub-studies were processed and accepted for publication quickly, nearly resulting in publication of these sub-studies prior to the main study being published first. *Lesson learned: Do not submit sub-studies for publication until the main study has been accepted for publication.* **Media:** Media outlets in the USA and Canada took interest in our study, resulting in investigators from various recruitment sties giving interviews to local and regional media outlets. *Lesson learned: Engage media at various recruitment sites to maximize dissemination.* **Translation to practice:** We wanted our study to inform the evidence review that the International Liaison Committee on Resuscitation (ILCOR) was conducting for 2015 resuscitation guidelines. Our study was published after the literature searches were conducted. Immediately after our study was published, we contacted the author of the question related to just-in-time training to ensure our study was included in the review process. *Lesson learned: Work with knowledge translation partners to determine their deadlines. Take knowledge translation efforts into consideration when developing research timelines.*

##### Simulation research pearl

Prior to proposing a research question, investigator(s) should complete a thorough review of the published literature within and outside of the simulation and/or healthcare domains. If a specific intervention has already been robustly studied in the clinical context, then there may be little reason to replicate the study in the simulated context.

#### Identifying outcome measures

Outcomes from simulation-based research have been described in the context of translational science, where T1 outcomes are those achieved in the simulation lab, T2 outcomes as those resulting in improved patient care practices, and T3 outcomes resulting in improvements in patient and public health [30]. Investigators should strive to measure T2 and T3 outcomes, which can be facilitated by partnering with outcomes centers and/or clinical researchers who are familiar with the processes involved in collecting clinical data. When collecting T2 or T3 outcomes is not feasible or applicable, T1 outcomes (knowledge, skills, and/or attitudes) can be captured in the simulated environment [2, 31, 32] (Table 1). If T1 outcomes are measured, it is important that the measurement tools used have sufficient validity evidence [33].

Utilizing tools that lack validity evidence place the results of studies in question. When validated tools are unavailable, investigators have the option of either modifying a pre-existing tool or developing a new one; either way, research should be done to describe the validity evidence supporting the use of the tool as an outcome measure [34, 35]. For example, when planning to conduct a study evaluating the impact of scripted debriefing for novice facilitators of a pediatric advanced life support course [12], we decided to measure learner knowledge and team clinical performance as outcomes. Prior to the main study, we developed and conducted a validation study for both the multiple-choice test [16] (i.e., knowledge) and the clinical performance tool (i.e., adherence to resuscitation protocols) [15]. Failure to invest time in gathering validation data for proposed outcome measures makes the results of the study difficult to interpret, with subsequent publication difficult to achieve.

##### Simulation research pearl

When simulation technology (e.g., mannequin or external device) is used to capture performance outcomes, investigators should ensure that the data captured is both reliable and accurate (Table 3). This may require standardized calibration of equipment across sites, collaboration with industry, and testing equipment at all sites prior to study implementation.

#### Pilot studies

Single-center pilot studies are the foundation for successful multicenter studies. Pilot studies help to inform potential modifications to the study question and study design, highlight challenges with protocol execution (e.g., obtaining consent, recruitment, data collection), and provide data to inform the power calculation of the sample size for the multicenter study [3, 27]. Protocol non-adherence or subject withdrawal can be captured to estimate the sample size for the multicenter study [36]. Lessons learned from pilot work should be integrated into the new multicenter study protocol, preventing potential issues from arising at that phase (Table 3). Single-institution pilot studies help to identify human and material resource needs that inform budgets. Sometimes, pilot data are noted from a review of published literature; investigators should contact first authors to explore the potential for collaboration and synergy.

##### Simulation research pearl

Do not underestimate the value of conducting pilot studies. Pilot data strengthens grant applications, particularly for multicenter simulation studies, where investigators can highlight how pilot work has identified solutions to simulation-specific research issues (e.g., standardizing scenario, blinding of reviewers, confederate training).

### Project development phase

#### Identify collaborators

Key collaborators for any multicenter study should have collective expertise in the relevant content area, clinical research, simulation research and/or education, study design, statistical analysis, technical expertise (if appropriate), and knowledge translation [37]. Team members may include principal and site investigators, research coordinators and assistants, a statistician, subject matter experts, simulation technicians, a psychometrician, trainee(s), and senior mentor(s). Involving trainees and junior faculty builds capacity for future studies, while mentors troubleshoot issues and provide career advice for early-career investigators [27]. In the planning phase, the principal investigator clarifies roles, responsibilities, expectations, and the projected time commitment for each team member. The principal investigator works with site leads to determine existing resources at each site, identifies opportunities for matching funds from institutions, and clarifies budget requests for future grant applications.

Central to the success of a multicenter research team is the establishment and maintenance of trust amongst investigators. Research team members must feel comfortable sharing ideas without fear of others stealing those ideas and turning them into projects or grants of their own. Establishing an agreement of shared confidentiality when the research team is first formed helps to lay the foundation for a trusting bond between investigators.

##### Simulation research pearl

Invite collaborators from outside of the simulation community—they often provide perspective that can help enhance study design, clinical applicability, and generalizability.

#### Protocol development

Protocol development is typically an iterative process involving feedback from diverse informed sources, revisions to the protocol, and consensus from investigators [3]. The initial draft should be discussed in a meeting with collaborators and, if possible, external experts who are not part of the study team.

Next, the study team typically requires several more planning meetings (in person, web-based, or conference calls) to reach consensus before finalizing the study protocol. Using internet-based file sharing and collaboration tools can facilitate ongoing asynchronous dialogue amongst collaborators [38]. Sometimes larger research teams run the risk of “analysis paralysis,” or spending too much time analyzing and debating the details of the study protocol without coming to consensus. If this occurs, it may help if a smaller core group of individuals develops the study protocol that is then circulated for final revision and approval by the entire research team.

##### Simulation research pearl

Investigators should present their study protocol to colleagues for peer review (Table 3). The INSPIRE network hosts a bi-annual meeting where investigators meet with collaborators and external experts to receive constructive feedback.

#### Research operations manual

The research operations manual provides a step-by-step guide, policy, and standard operating procedures for the execution of the study protocol [39]. The manual should be organized to provide the necessary information for a collaborator at any site to recruit subjects and collect data; including study team members, study flow, inclusion and exclusion criteria, study methods, confederate training, and details regarding data collection and sharing. Listing members with appropriate contact information ensures that collaborators have someone to contact for support and guidance.

Data management procedures are critical to a successful multicenter study. A centralized method of collecting data (e.g., online website, centralized database) allows for data to be entered remotely (i.e., either by collaborator and/or study participants), which is particularly important for multicenter research [40, 41]. Data security and privacy concerns are heightened if data can be entered or viewed by investigators across institutions [42]. The principal investigator determines the level and degree of access for each team member. The manual also describes the unique identification system for each study participant (or team), while providing a means of identifying data across nested factors such as time points, recruitment sites, and intervention groups [42]. Standard procedures for data abstraction, verifying the accuracy of data, and generation of data backups should be described within the manual.

The ability to isolate the independent variable by minimizing the influence of other variables is an advantage of simulation research; the challenge is how to accomplish this across multiple sites (Table 1). Participants should be oriented to the technology and the environment in a standardized fashion. When confederates (i.e., actors) are used in SBR, they should be trained and monitored to ensure consistent performance across sites (Table 3) [43]. In prior work, we describe how the use of cue cards, online learning, and videos modeling ideal confederate behavior result in highly consistent confederate performance in a multicenter trial [43]. The same type of simulator (e.g., task trainer, mannequin) should be used across all recruitment sites, and the simulated environment should be set up in the same manner to reduce potential confounders. Standardizing the simulation event, including clear learning objectives, use of adjuncts and facilitator characteristics should be discussed when preparing the protocol. All relevant elements of instructional design (e.g., duration, timing, frequency, clinical variation, assessment, adaptability, range of difficulty, adjuncts, integration, feedback/debriefing) need to be considered—for educational studies, these represent significant confounding variables that can be minimized through careful planning and feasibility testing.

##### Simulation research pearl

A detailed description of how the protocol is standardized across sites helps to minimize risk of bias [1]. Recently published reporting guidelines describe important standardization elements, including participant orientation, simulator type, simulation environment, simulation event/scenario, instructional design, and feedback and/or debriefing [44–47].

#### Grant preparation

Grant support is often necessary to conduct a multicenter research project [48]. The project team identifies candidate funding agencies, which may vary depending upon research focus and country of origin. We recommend applying to multiple opportunities (if permitted by funding agency) to maximize chances of securing funding. Smaller grants present opportunities to fund pilot, validation work, or portions of the main study. Grants should include preliminary data from pilot studies and should be written to highlight the strengths of the research team and prior collective successes. Rejected grants with reviewer comments should not be viewed negatively; rather, they offer feedback that can improve future grant submissions.

When preparing a budget, the principal investigator should request institutional budgets from each site investigator (to inform the larger budget), identify opportunities for matching or in-kind funds from collaborating institutions, and determine which research positions will provide best value for money. We recommend prioritizing some funding to support network infrastructure, as money goes much further when allocated to site research coordinators/assistants than it does in purchasing equipment or protecting investigator time. Management of the overall budget and distribution of funds is typically the responsibility of the principal investigator (lead research site). Additional administrative support should be allocated to the lead research site in the budget to account for management of finances. Sometimes, enthusiasm at a site wanes, resulting in unfulfilled commitments. To manage this issue, one or two backup sites can be identified in the grant proposal. These sites assist with recruitment if other sites are falling short. The responsibilities of each institution should match the funding allocation with a transparent process of sharing the budget across centers.

Ultimately, some projects may either be left unfunded or underfunded. If so, the research team needs to determine if the project can be feasibly completed with existing infrastructure. In our experience, multicenter research projects can be completed with fairly modest budgets if the principal and site investigators are passionate, enthusiastic, and have the time and energy to fully commit to the project.

##### Simulation research pearl

Grant reviewers may criticize studies with simulation-based T1 outcomes. To highlight the clinical relevance of these studies, investigators should attempt to describe potential links between proposed outcomes, other accessible outcomes, and patient outcomes (Table 1). Doing so provides a chain of causality that may strengthen the rationale for the study [49].

#### Ethical approval and subsite contracts

Obtaining institutional review board (IRB) approval can be a rate-limiting step for a multicenter research study. IRBs at different institutions may have varying requirements and a range of review levels, from exempt to review by the full board [50]. To reduce workload and streamline the IRB process, principal investigators should prepare and circulate a copy of the approved IRB submission with accompanying documentation (e.g., protocol, consent forms, demographic forms, assessment tools) that can be used as a template for other site investigators (Table 1). We have found that a presentation (via webinar or face to face) given by the principal investigator describing elements of the IRB submission helps engage collaborators who are preparing their own IRB submissions. Enlisting the assistance of an IRB staff member can be very helpful when navigating multicenter and particularly multinational IRBs.

Subsite contracts between the primary investigators’ institution and site investigator institutions are typically required if data and/or money is being transferred between sites. Contracts help to ensure participant (i.e., learner, provider or patient) confidentiality across all sites and outline data sharing and financial agreements. Typically, contracts cannot be executed until ethical approval has been obtained at both sites. These legal contracts can take many months to complete and can cause lengthy delays in research. Obtaining IRB approval early in the planning phase and quickly moving onto subsite contracts can help keep teams on track.

##### Simulation research pearl

Multicenter research teams can manage the variability with IRBs across institutions [51] by sharing IRB comments with collaborators so that issues can be addressed in future submissions or amendments. Some IRBs will allow for a ceded review for simulation studies, either on a case-by-case basis or as a pre-approved agreement [52].

#### Clinical trial registration

Clinical trial registries are “web-based databases providing researchers, journal editors, and reviewers detailed study information to help inform trial results” [53], with the expressed intent to determine the degree of publication bias. While the International Committee of Medical Journal Editors (ICMJE) requires that clinical trials be registered prior to publication in their member journals, there is controversy over whether simulation-based studies should also meet this requirement (Table 1). The ICMJE definition suggests that registration is necessary for studies examining the effect of providers on patients (i.e., T1 outcome), but not necessary for studies examining the effect of the providers (i.e., T2 or T3 level outcomes) [53]. Our experience has been variable, with most top medical journals requesting clinical trial registration numbers prior to considering studies for publication.

##### Simulation research pearl

We recommend prospectively registering simulation-based studies, thereby eliminating the possibility that a manuscript be rejected due to a lack of clinical trial registration (Table 3) [53].

#### Manuscript oversight committee

The manuscript oversight committee (MOC), typically comprised of three members, is tasked with ensuring academic rigor, transparency for authorship assignment, and managing conflict of interests for potential publications [8]. The MOC works with the primary investigator to list all potential publications and then generates writing teams for each publication in a fair and logical manner. Key authorship positions are allocated based upon projected degree of involvement and workload, while also providing opportunity for novice investigators to serve in key writing roles. Developing and sharing an MOC document during the planning phase of research provides investigators opportunity to give feedback on proposed writing teams and prevents conflict between team members (Table 3). See Additional file 1 for a sample MOC document.

##### Simulation research pearl

Simulation studies can be published in many different journal types (e.g., simulation journals, healthcare education journals, clinical journals). Research teams should consider the journal scope, focus, and audience when selecting a journal—and matching this to the study topic, quality, and ideal audience (Table 1).

#### Feasibility testing

As one of the final steps of the project development phase, feasibility testing serves as an important test to determine if all sites are properly prepared to execute the study protocol. During feasibility testing, all sites should conduct several study rehearsal sessions using volunteers that would not be typically eligible to participate in the study. The sessions can serve multiple purposes: to train research personnel and test the technical aspects of research, including mannequin operation, audio/video capture (if applicable), and data collection/sharing (Table 1). If audio and video from one or more angles is being used to capture outcomes, we recommend that each site send audio/video from feasibility testing sessions to the primary investigator for review and approval before commencing recruitment. Test data should be uploaded to the database to ensure that data collection system is functional.

##### Simulation research pearl

The simulation environment across research sites may be variable. When reviewing videos from feasibility testing, pay close attention to the simulation environment to identify possible sources of variance across sites (Table 3).

### Study execution phase

#### Recruitment and enrollment

Clear and concise inclusion and exclusion criteria ensure participant types are similar across all sites. For randomized controlled trials, block randomization ensures equal allocation of study groups within study sites. Ideally, the randomization process should occur centrally (e.g., randomization envelopes created at one site), thus eliminating the chance for randomization to occur differently between sites.

##### Simulation research pearl

Individuals from various sites may have different expectations of participation in simulation-based research and may wonder how it differs from simulation-based education and/or assessment (Table 1). To avoid confusion and systematic bias, the consent process can be standardized by using a video to introduce the study, potential risks, and how the session(s) may differ from simulation-based education.

#### Communications and decision-making

Clear communication between the principal investigator and collaborators prevents challenges from arising and keeps the research project on the proposed timeline. A research steering committee comprised of the primary investigator, site co-investigators, and administrative support should meet regularly by conference call and annually in person to review progress and make key decisions [37]. Individual sites will have their own research operations committees, comprised of research assistants, coordinators, and the site investigator, to discuss recruitment and to troubleshoot any local issues. Establishing a clear organizational structure, along with a shared goals and expectations, creates a team-based research environment where individuals buy into their role as a collaborative team member [54].

Sometimes despite having an established organizational structure, site investigators may lose interest in the study or be pulled away by competing priorities [27]. To manage this issue, the primary investigator must foster a collaborative spirit by building team cohesion (e.g., team dinners at conferences), celebrating successes (e.g., first participant recruited, presentations, publications), and providing positive feedback. Understanding the areas of expertise of site investigators allows the primary investigator to assign responsibilities that are most likely to fully engage the team member. We have found that quarterly newsletters and site performance dashboards (e.g., to report recruitment numbers) to be effective motivating tools during the study execution phase of multicenter studies.

##### Simulation research pearl

Simulation fellowship training programs offer opportunity to engage trainees in multicenter research. A training committee comprised of the primary investigator, trainee(s), and their supervisor(s) should be established to oversee trainee progress and academic productivity.

#### Quality assurance

Primary investigators should work with the research team to implement a quality assurance plan to prevent, detect, and address problems as they arise. In the study execution phase, detection of problems via routine monitoring can be accomplished through centralized monitoring of data (e.g., automated data field screening), centralized review of videos, and site visits [55]. Centralized monitoring of data allows for detection of missing or incorrect entry of specific data points.

A schedule of site visits by the principal investigator or core study team should be developed, ideally timed to correspond with the initiation of recruitment, and near the middle of the trial if funding permits. The initial site visit may be used as an opportunity to train research staff and confederate actors (if applicable) [43]. The individual conducting the site visit reviews the research operations manual with the local study team, reviews recruitment and data entry procedures, and addresses any pressing concerns [55]. Mid-trial site visits includes the above plus on-site review of local study data to identify any possible issues. If errors or systemic issues are detected, the primary investigator many need to (a) revise the study protocol to prevent future errors, (b) retrain research staff, (c) conduct future audits, and (d) report on protocol violations in publications [55].

##### Simulation research pearl

Intermitted centralized review of videos can identify issues with video and audio quality, adherence to blinding of participants, or deviations in confederate behaviors that may affect study outcomes (Table 3).

#### Data abstraction and analysis

Rater orientation training is required when assessment tools are used to collect performance data [56, 57]. Rater orientation training ensures all raters have a shared understanding of the construct(s) being assessed and provides opportunity to calibrate raters immediately prior to data abstraction [1]. If raters are expected to abstract data at multiple points during or after the study, then booster training (or re-training) should be offered to re-calibrate raters prior to each assessment time point (Tables 1 and 3). Online website and centralized databases can be used to collect and assign videos to raters, who can then submit ratings in an asynchronous fashion [40].

Multicenter SBR is prone to missing data. Missing data should be analyzed to determine if systematic biases exist (e.g., poor performances are not captured, data missing from one site only). In multi-center trials, some of the variation in the outcome may be at the institution level—this is explicitly true in cluster-randomized trials—which should be taken into account both to ensure proper analyses and as a phenomenon of considerable interest.

##### Simulation research pearl

When assigning videos to raters, investigators should attempt to avoid allocating videos of participants to raters from the same site. While raters may be blinded to the intervention, recognizing individuals who are their colleagues and/or trainees may introduce bias to the rating process.

### Dissemination phase

#### Presentation and publication

**Fig. 2:**
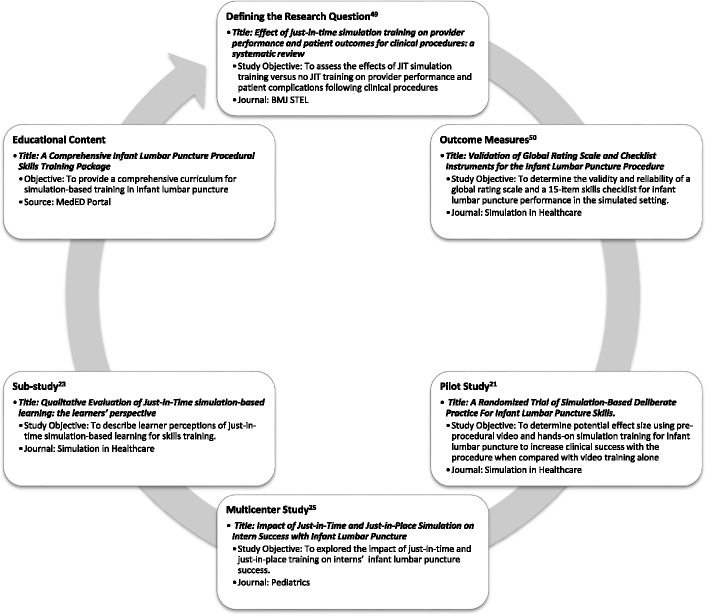
Opportunities for publication during multicenter research

Once data collection and analyses have taken place, the writing phase begins for professional scholarly output in the form of presentations and publications. Abstracts can be submitted for presentation at multiple conferences (if conference guidelines permit) to promote greater dissemination. Publications should be prepared according to the MOC document and formatted by following reporting guidelines for healthcare simulation research [44–46]. Following the steps outlined in this paper should offer various opportunities for publication, including systematic or narrative reviews (i.e., defining the research question), assessment tool validation studies (i.e., outcome measures), pilot studies, the main multicenter study, associated sub-studies, and educational content (if applicable). Fig. 2 offers an example of a series of publications resulting from work-related to a multicenter study examining the impact of just-in-time lumbar puncture training [21, 23, 25, 58, 59]. Publishing the systematic or narrative review, validation study, and pilot study early allows for citation of this work in the main multicenter study paper. Similarly, the multicenter manuscript should be published ahead of associated sub-studies (Table 3). Sub-studies should be planned ahead of time to ensure they address novel objectives and report outcomes that do not completely overlap with those reported in the main study.

##### Simulation research pearl

Educational interventions developed for simulation-based educational studies can be disseminated through publication to facilitate implementation by educators (e.g., meded portal) (Table 1).

#### Media

Although embargo and copyright rules from journals may prevent disseminating findings prior to publication, the research team should carefully strategize how to best disseminate the knowledge through traditional media (e.g., television, newspaper), social media, webinars, podcasts, and/or blogs once results have been published. “Free Open Access Meducation” (FOAM) in the form of blogs, podcasts, and associated social media strategies have seen increasing uptake in the healthcare community as a means of disseminating new research to the masses [60–62].

##### Simulation research pearl

Engaging the editors of simulation-focused websites (e.g., www.simulationpodcast.com, www.debrief2learn.org) may facilitate dissemination in the form blogs, podcasts, or online article reviews. These sites discuss new research and engage the simulation community in online discussion of recently published work.

#### Translation to practice

Dissemination of research is incomplete without engaging relevant stakeholders in efforts to translate results to practice. Collaboration with existing organizations that share similar goals can enhance dissemination (Table 3). For example, after completing a multicenter study on scripted debriefing [12], our research team collaborated with the American Heart Association to integrate a scripted debriefing tool into new instructor training materials for advanced life support courses [63]. Similarly, procedural skills studies conducted by INSPIRE investigators have spawned a collaboration with Open Pediatrics, with the goal of producing procedural skills training kits for residents and practicing physicians. Lastly, sites within simulation networks provide an established and receptive dissemination conduit for uptake of new research findings, serving as a powerful knowledge translation vehicle for completed multicenter studies.

##### Simulation research pearl

Begin with the end in mind. Know ahead of time who your key stakeholders are and engage them in defining the research question, study design, and protocol development. This will help to maximize the likelihood of uptake and dissemination once the study is completed.

## Conclusion

The conduct of high-quality multicenter simulation-based research is challenging. Success may be enhanced by following a stepwise approach including four distinct phases of multicenter research: Planning, Project Development, Study Execution, and Dissemination. While a stepwise approach offers structure to formalize the research process, multicenter collaboration is often not completely linear in nature. Deliberate, thoughtful and collaborative decision-making occasionally requires the need to cycle back and revisit a step or two in the research process. These mini feedback loops facilitate the maintenance of a shared mental model amongst investigators, which is a critical element of successful collaborative research. We hope this guidance will encourage investigators to conduct multicenter research, and in doing so, advance the rigor and quality of SBR.

## Additional file

Additional file 1: Sample Manuscript Oversight Committee Document. (DOC 44 kb)

## Abbreviations

ICMJE: International Committee of Medical Journal Editors
INSPIRE: International Network for Simulation-based Pediatric Innovation, Research and Education
IRB: Institutional review board
MOC: Manuscript oversight committee
SBR: Simulation-based research
