# Supplementary material for: Conducting multicenter research in healthcare simulation: Lessons learned from the INSPIRE network
Source: Adv Simul (Lond). 2017 Feb 28;2:6. doi: 10.1186/s41077-017-0039-0 (PMC5806260; doi:10.1186/s41077-017-0039-0)
Supplement: Supplementary file 1 — Sample Manuscript Oversight Committee Document. (DOC 44 kb) [file 41077_2017_39_MOESM1_ESM.doc]

**Additional File 1**

**Sample Manuscript Oversight Committee Document**

**INSPIRE QCPR Research Group**

**Manuscript Oversight Committee (MOC)**

**General Principles**

1. All site investigators will be recognized as part of the “INSPIRE QCPR Investigators” in all project-related publications with the byline *"...FOR THE INSPIRE QCPR INVESTIGATORS."* provided they meet ICMJE requirements for authorship.
2. Local INSPIRE QCPR investigators at sites who are not site PI's or co-PI's will generally be acknowledged in an appendix of site participants appropriate to that manuscript (this will include acknowledgement of research coordinators, local investigators or organizers of research)
3. All site investigators will be recognized with named authorship in at least 1 INSPIRE QCPR publication
4. First, second, third and senior authorship will be assigned to individuals who have done the most amount of work (ie. development and organization of protocol or tool, recruited many subjects, etc) for that specific project/manuscript. These, for the most part, are individuals who have been working on the project over the past 2-3 years, and who have been involved from the inception of the project.
5. Prioritize young investigators as first, second and third authors, when possible and appropriate.
6. Attempt to appropriately attribute academic credit, with emphasis on acknowledgement of all involved (usually with most input from first and senior authors as to order)
7. MOC subcommittee to negotiate and mitigate disagreements among/between investigators about authorship
8. Facilitate identification and management of Conflicts of Interest, if they arise
9. Assist with enforcement of timelines for analysis and publication of data. If any first author cannot complete the manuscript submission within 1 year following the end of data collection/closure of data collection, then first authorship will be re-evaluated and potentially reassigned by the MOC.

**Proposed Manuscripts (2014)**

*Code* : **Bold = First Author**; **Blue = 2^nd^/3^rd^ Author**; **Red = Senior Author**; Named contributing authors

1. **Assessing and Improving CPR with CPRcard and Video-based Learning**
   1. Objective : Evaluate the effect of video-based learning with CPRcard and use of CPRcard during simulated pediatric cardiac arrest in pediatric healthcare teams.
   2. Target Journal : **JAMA Pediatrics**
   3. Writing Group : **AAA**, **XXX, YYY**, **ZZZ**
   4. Additional Writing Group:
   5. ...for the INSPIRE QCPR Investigators
2. **Perceptions of CPR – Quality of CPR as perceived by team member and team leader**
   1. Objective: Describe the perceptions of quality of CPR of team members and team leader as compared to data collected by CPRcard
   2. Target Journals: Resuscitation, Pediatrics
   3. Writing Group : **AAA**, **XXX, YYY**, **ZZZ**
   4. Additional Writing Group:
   5. ...for the INSPIRE QCPR Investigators
3. **Variability of CPR Quality Across Sites and Influence of JIT training and Feedback**
   1. Objective: Compare the quality of CPR across sites and describe the influence of JIT training and feedback
   2. Target Journal: Resuscitation, PCCM, Annals of Emergency Medicine
   3. Writing Group : **AAA**, **XXX, YYY**, **ZZZ**
   4. Additional Writing Group:
   5. ...for the INSPIRE QCPR Investigators
4. **Task Load during Pediatric Resuscitation (Team Leader vs Team Member)**
   1. Objective: Describe and compare the perceptions of task load for team members and team leaders during pediatric cardiac arrest
   2. Target Journal: PCCM, SiH Journal
   3. Writing Group : **AAA**, **XXX, YYY**, **ZZZ**
   4. Additional Writing Group:
   5. ...for the INSPIRE QCPR Investigators

**Proposed Manuscripts (2015)**

*Code* : **Bold = First Author**; **Blue = 2^nd^/3^rd^ Author**; **Red = Senior Author**; Named contributing authors

1. **Qualitative Study – Variables Influencing the Quality of CPR During Pediatric Cardiac Arrest**

- 1. Objective: Describe the various factors that influence the quality of CPR, and describe various themes that emerg from qualitative interviews
  2. Target Journal: Resuscitation, PCCM
  3. Writing Group : **AAA**, **XXX, YYY**, **ZZZ**
  4. Additional Writing Group:
  5. ...for the INSPIRE QCPR Investigators

1. **Confederate Compliance Substudy**
   1. Objective : Describe methodology of confederate training and report compliance rate
   2. Target Journal : SSH Journal
   3. Writing Group : **AAA**, **XXX, YYY**, **ZZZ**
   4. Additional Writing Group:
   5. ...for the INSPIRE QCPR Investigators
